# Trend and projection of non-communicable diseases risk factors in Iran from 2001 to 2030

**DOI:** 10.1038/s41598-024-58629-z

**Published:** 2024-04-06

**Authors:** Farshad Farzadfar, Moein Yousefi, Ali Jafari-Khounigh, Zahra Khorrami, AliAkbar Haghdoost, Fatemeh Khosravi Shadmani

**Affiliations:** 1 Non-Communicable Diseases Research Center, Endocrinology and Metabolism Population Sciences Institute, Tehran University of Medical Sciences, Tehran, Iran; 2 Department of Mathematics and Statistics, Memorial University of Newfoundland, St John’s, NL Canada; 3 Road Traffic Injury Research Center, Tabriz University of Medical Sciences, Tabriz, Iran; 4 Ophthalmic Epidemiology Research Center, Research Institute for Ophthalmology and Vision Science, Shahid Beheshti University of Medical Sciences, Tehran, Iran; 5 HIV/STI Surveillance Research Center, and WHO Collaborating Center for HIV Surveillance, Institute for Futures Studies in Health, Kerman University of Medical Sciences, Kerman, Iran; 6 Research Center for Environmental Determinants of Health (RCEDH), Health Institute, Kermanshah University of Medical Sciences, Kermanshah, Iran

**Keywords:** Trend, Projection, Non-communicable diseases, Risk factors, Iran, Risk factors, Health care, Health policy

## Abstract

This study aims to investigate the trends and project the major risk factors of Non-communicable Diseases (NCDs) in Iran. We obtained the trend of prevalence of main risk factors related to NCDs in 30 to 70-year-old-individuals. The data were extracted from WHO STEP wise approach to NCDs risk factor surveillance (STEPS) survey. Also,the previous studies conducted at national and subnational levels from 2001 to 2016 were employed. The prevalence of risk factors was projected by 2030 using Bayesian Model Averaging (BMA) and Spatio-temporal model stratified by sex and province. The percent change for the age-standardized prevalence of smoking in men between 2001 and 2016 was calculated to be − 27.0. Also, the corresponding values for the risk factors of diabetes, hypertension, obesity and overweight, physical inactivity (PI), and mean of salt intake were − 26.1, 29.0, 70.0, 96.8, 116.6, and 7.5, respectively. It is predicted that smoking and these risk factors will undergo a change to show values of − 1.26, 38.7, 43.7, 2.36, and 15.3 by 2030, respectively. The corresponding values in women for the time interval of 2001–2016 were − 27.3, 26.3, 82.8, 1.88, 75.2, and 4.2, respectively. Plus, projections indicate that the 2030 variation values are expected to be − 25.0, 16.7, 37.5, 28.7, 26.7, and 10.9 respectively. This study showed that the prevalence of four risk factors of PI, overweight and obesity, hypertension, and diabetes is increasing in Iran. Therefor, it is necessary to carry out effective interventions to adopt a healthy lifestyle and reduce the risk factors.

## Introduction

Due to the demographic and epidemiological transition in the world, mortality from infectious diseases has been reduced and non-communicable diseases (NCDs) are, now, considered to be a global health priority. In 2019, NCDs were responsible for the death of 42 million people, which is equivalent to 74% of all deaths globally^1^. Furthermore, NCDs contribute to 63% of the disability-adjusted life years (DALYs)^1^.

The burden of NCDs is unequally distributed among countries, low and middle income countries (LMICs) having a different experience from high income countries. Notably, 77% of all NCDs deaths are in LMICs, and these countries are expected to see a 17% increase in NCDs-related deaths within the next decade^2^. On the other hand, the median age of onset for NCDs is falling globally. Each year, over 15 million people aged 30–70 die from a NCDs with 85% of these "premature" deaths occuring in LMICs^3^. However, the target 3.4 of the Sustainable Development Goals (SDGs) aims to reduce premature mortality from NCDs by one third by 2030^4^.

According to the World Health Organization, non-communicable diseases in Iran, a middle-income country, are the leading cause of mortality, contributing to 75.3% of the overall disease burden in the country^3^. The main causes of deaths from NCDs are categorized into four categories (cardiovascular disease, cancers, diabetes and asthma, and COPD)^5^.

The basic element to management and control of NCDs lies in focus on primary prevention and reducing the prevalence of modifiable risk factors^6^. Major modifiable risk factors for NCDs include smoking, high salt intake, high blood pressure, high fasting blood glucose, and high Body Mass Index (BMI)^7^. It is estimated that 40% of NCDs deaths are caused by the consumption of saturated and trans-fat, salt and sugar^8^. Additionally, salt consumption is responsible for 30% of high blood pressure cases^9^, and 8% of NCDs deaths are attributed to high blood pressure^10^.

Understanding the trend and projection of future of risk factors is crucial for controlling and managing the prevalence of NCDs and designing appropriate preventive interventions. The aim of this study is to investigate the trend and project the major risk factors of NCDs at national and subnational levels in Iran. Thus far, no study in Iran has investigated and projected the major NCD risk factors to inform the design of appropriate preventive interventions at national and subnational levels.

## Methods

### Overview

We obtained the trend of prevalence of common risk factors related to NCDs (smoking, PI, mean of salt intake, hypertension, obesity and overweight, and diabetes) in 30 to 70 year-old individuals. In addition, we used data from STEPs surveys and prior national/subnational studies (2001–2016). Plus, the prevalence of risk factors was projected to 2030 using a spatiotemporal model and Bayesian Model Averaging (BMA) stratified by sex and province.

### Study location

Iran, a middle-income country located in Western Asia, is bordered by Iraq, Turkey, Azerbaijan, Armenia, Turkmenistan, Afghanistan and Pakistan. It covers an area of 1,648,195 km^2^, making it the fourth-largest country entirely in Asia and the second-largest country in Western Asia. The current population of Iran is 89,348,412 based on projections of the latest United Nations data in 2023^11^. According to the latest World Bank data published in 2020 life expectancy at birth in Iran is 76.78 years^12^. Iran is subdivided into thirty-one provinces which are different economically, culturally and climatically (https://www.amar.org.ir).

### Data sources

#### Risk factors

This study was conducted based on the STEPwise approach to NCDs risk factor surveillance (STEPs), with previous studies conducted at national and subnational levels (see Appendix 1, Table 1). The STEPs survey was carried out in 2005, 2006, 2007, 2008, 2009, 2011 and 2016, with a large sample size to represent the Iranian population (detailed sample sizes provided in Appendix 1, Table 2). Additional studies, with details listed in Appendix 1, Table 1, were employed to estimate the prevalence of diabetes, hypertension, obesity, and overweight. The definition of risk factors is presented in Table 1. Missing data points for the prevalence of risk factors were interpolated and extrapolated using Gaussian process regression (GPR) and spatiotemporal models. Further details are available elsewhere^13–16^. Risk factor prevalence for individuals aged 30 to 70 years was standardized using a direct approach with the 2015 population and estimated according to sex subgroups.

**Table 1:** Definition of risk factors.

Risk factors	Definition
Smoking	Proportion of the population who are reporting to smoke every day
Diabetes	Fasting glucose plasma ≥ 126 mg/dL or (7 mmol/L)
Hypertension	High blood pressure means of systolic ≥ 140 or diastolic blood pressure ≥ 90 mmHg or being under treatment with blood pressure drug
Overweight and obesity	Body mass index ≥ 25 (kg/m^2^)
Physical inactivity	Average weekly physical activity at work, home, transport-related, and recreational measured by MET minutes per week. Physical inactivity as defined METs less than 600 (METs-minutes/week)
Salt intake	Salt intake 24 h urinary sodium measured in gram per day

**Table 2:** Percent of change of risk factors by sex and province.

Province	Sex	Risk factors											
		Smoking		Physical inactivity		Salt intake		HTN		Obesity and overweight		Diabetes	
		2001–2016	2016–2030	2001–2016	2016–2030	2001–2016	2016–2030	2001–2016	2016–2030	2001–2016	2016–2030	2001–2016	2016–2030
Markazi	F	– 29.1	– 28.2	84.5	29.6	– 2.0	– 5.2	92.3	40.9	91.8	28.7	38.9	25.0
	M	– 25.1	– 24.2	142.9	40.7	– 14.3	– 21.6	75.4	43.8	101.0	44.6	50.0	27.6
Gilan	F	– 30.3	– 30.4	83.4	28.9	5.8	5.5	104.8	40.5	80.3	25.4	34.7	25.7
	M	– 27.8	– 25.4	138.3	39.7	– 6.8	– 11.5	91.9	45.1	91.7	40.7	45.8	30.2
Mazandaran	F	– 31.4	– 28.6	70.2	24.8	– 15.9	– 34.4	90.7	36.7	73.9	22.5	17.7	13.3
	M	– 29.5	– 28.0	104.1	33.7	– 24.0	– 54.7	71.3	37.4	82.4	35.6	17.9	13.0
Azerbaijan_East	F	– 25.8	– 26.1	57.8	20.9	– 6.3	– 25.8	74.7	34.1	84.2	26.6	24.4	17.6
	M	– 26.9	– 26.5	79.8	28.1	– 16.5	– 38.5	69.1	38.0	91.6	41	28.4	19.8
Azerbaijan_West	F	– 23.5	– 23.1	103.3	33.9	– 19.7	– 40.4	83.6	36.6	84.7	25.9	34.2	23.5
	M	– 26.3	– 25.4	198.4	46.7	– 23.4	– 44.9	66.9	35.9	98.2	39.8	37.9	21.3
Kermanshah	F	– 22.4	– 23.7	87.8	30.4	8.2	6.7	91.1	38.9	87.5	27.2	31.2	20.8
	M	– 26.7	– 24.5	153.0	41.4	9.4	6.7	76.3	40.7	96.0	42.3	30.6	19.8
Khuzestan	F	– 25	– 25.6	78.5	27.6	0	– 5.4	90.9	39.7	85.9	27.9	18.8	12.5
	M	– 27.5	– 28.4	126.9	38.4	– 8.5	– 16.5	71.5	39.8	94.9	43.5	19.3	12.1
Fars	F	– 26.1	– 26.5	160.4	42	3.3	– 5.4	68.8	33.3	96.7	32.9	20.7	15.2
	M	– 27.9	– 27.2	259	63.1	– 6.6	– 17.2	70.4	37.4	94.9	44.7	22.7	14.8
Kerman	F	– 25.7	– 26.9	97.7	32.3	– 18.5	– 48.9	66.5	32.8	102.3	36.4	16.7	7.8
	M	– 25	– 23.4	180.1	44.6	– 23.3	– 59.8	54.8	32.5	104.7	51.1	18.2	6.2
Khorasan_Razavi	F	– 26.2	– 22.6	110.5	35.2	– 5.9	– 13.5	82.7	37.5	96.8	32.7	34.4	20.9
	M	– 27.2	– 25.7	232.1	49.4	– 3.0	– 4.1	71.7	37.2	104.9	47.6	34.0	19.7
Isfahan	F	– 21.6	– 20.7	62.1	22.6	0.0	– 18.9	81.3	37.7	84.2	28.2	22.4	19.2
	M	– 21.7	– 19.9	88.8	30.3	– 1.9	– 19.8	81.7	40.8	95.8	43.1	27.2	19.4
Sistan & Baluchistan	F	– 21.7	– 22.2	86.3	29.7	– 10	– 16.7	86	43.1	110.3	41.8	34.9	22.4
	M	– 25.3	– 23.8	146	40.2	– 13.4	– 20.6	72.3	40.4	115.7	54.5	30.8	19.1
Kurdistan	F	– 23.3	– 21.2	97.7	32.7	– 8.9	– 21.6	107.4	43.2	94.3	29.7	49.2	28.7
	M	– 28.8	– 28.7	182.7	44.8	– 2.8	– 18.3	86.0	42.9	102.2	42.9	48.0	25.7
Hamadan	F	– 27.3	– 28.1	63.5	23.3	– 10.8	– 26.3	97.7	38.8	97.0	30.6	32.3	19.8
	M	– 28.7	– 28.3	91.4	30.8	– 19.2	– 37.6	67.3	37.4	98.9	44.0	30.2	18.8
ChaharM & Bakhtiari	F	– 27.5	– 27.6	60.8	22.0	2.0	– 12.0	82.6	36.6	91.7	30.6	21.7	13.1
	M	– 26.5	– 25.7	86.8	29.8	– 4.8	– 26.3	62.7	34.2	102.3	45.5	32.1	18.6
Lorestan	F	– 27.5	– 24.1	61.0	21.6	– 8.6	– 25.0	100.6	42.5	98.8	30.2	30.3	19.8
	M	– 25.8	– 24.3	85.2	29.3	– 11.3	– 26.5	66.4	39.5	104.7	46.0	34	19.7
Ilam	F	– 27.9	– 25.8	76.3	26.7	– 12.5	– 23.1	124.8	49.8	100.7	33.4	35	22.2
	M	– 29.1	– 28.4	118.1	36.2	– 16.8	– 23.4	88.6	43.5	101.3	47.0	40.4	24.2
Kohgiluyeh & BoyerAhmad	F	– 27.1	– 23.3	66.6	24.0	– 16.2	– 35.5	83.7	38.9	90.0	28.1	63.3	35
	M	– 30.2	– 27.9	98.4	32.7	– 20.8	– 50.5	73.1	40.2	105.4	42.9	70.3	33.3
Bushehr	F	– 28.9	– 28.1	58.5	21.2	– 20.0	– 47.6	62.4	29.8	91.8	31.7	20.0	14.3
	M	– 28.7	– 27.9	81.1	28.5	– 25.0	– 58.6	52.3	32.2	100.0	46.3	41.5	32.0
Zanjan	F	– 25.5	– 28.6	68.4	25.0	– 11.6	– 33.3	82.1	35.9	93.2	29.4	40.3	21.8
	M	– 27.0	– 27.6	100.8	33.3	– 14.8	– 34.6	61.9	33.9	101.1	44.9	51.0	24.3
Semnan	F	– 31.4	– 29.2	74.6	26.8	– 2.1	14.7	86.5	37.2	83.2	27.6	15.6	10.8
	M	– 27.1	– 24.5	117.5	36.2	– 1.0	9.4	78.1	38.4	99.0	43.1	19.5	14.3
Yazd	F	– 29.3	– 31.0	91.0	30.9	1.1	19.1	79.6	36.6	83.8	27.6	13.6	8.8
	M	– 30.3	– 30.6	159.7	42.6	– 4.8	9.0	80.7	41.3	90.0	42.1	13.0	6.7
Hormozgan	F	– 28.6	– 26.7	101.6	33.6	– 10.0	– 23.5	87.2	38.0	96.2	34.1	33.3	22.4
	M	– 28.3	– 26.3	201.6	46.7	– 9.8	– 21.7	80.6	40.5	108.1	48.8	34.8	21.0
Tehran	F	– 24.2	– 20.0	73.8	26.2	0.0	– 6.5	69.2	34.5	74.4	24.9	14.1	10.5
	M	– 20.1	– 17.1	110.4	34.9	– 4.0	– 11.3	74.3	38.8	80.0	37.7	21.3	16.5
Ardabil	F	– 26.7	– 27.3	94.1	31.5	– 6.1	– 18.5	91.3	38.9	73.6	23.8	37.9	22.0
	M	– 29.7	– 28.7	166.4	43.2	– 4.2	– 25.0	72.2	43.4	87.7	38.4	44.2	25.3
Qom	F	– 25.6	– 28.1	78.2	27.5	– 5.5	– 20.3	86.5	39.1	81.4	25.7	12.9	9.2
	M	– 24.1	– 25.8	122.6	37.1	– 7.9	– 15.9	78.7	41.4	93.8	41.0	14.9	9.3
Qazvin	F	– 25.0	– 23.1	82.1	29.0	– 11.5	– 28.3	88.9	38.8	86.6	28.5	30.1	20.4
	M	– 22.6	– 19.5	133.9	39.1	– 9.8	– 19.8	70.7	39.3	98.0	44.3	35.7	23.2
Golestan	F	– 28.6	– 25.7	93.0	31.5	9.0	4.1	94.2	41.9	95.2	27.4	24.7	17.6
	M	– 30.4	– 29.1	166.2	43.4	3.1	4.0	85.6	49.6	84.0	38.8	24.6	14.1
Khorasan_North	F	– 27.5	– 27.6	45.2	14.0	14.0	20.4	89.6	36.9	91.3	32.5	37.5	26.0
	M	– 32.0	– 30.8	57.7	20.6	9.2	14.0	73.3	36.9	119.5	49.1	38.3	21.5
Khorasan_South	F	– 29.5	– 29.0	60.2	21.9	3.4	– 7.7	90.8	42.8	97.7	38.2	35.1	23.4
	M	– 33.4	– 31.1	85.2	29.3	– 2.9	– 18.0	69.3	37.6	112.9	51.5	31.9	19.4
Alborz	F	– 26.2	– 25.8	61.1	22.0	6.7	23.2	41.3	15.2	70.7	25.1	12.5	9.4
	M	– 23.0	– 21.3	85.1	29.7	2.0	13.7	43.8	19.6	97.2	40.7	22.4	18.3
Iran	F	– 27.3	– 25.0	75.2	26.7	– 4.2	– 10.9	82.8	37.5	88.1	28.7	26.3	16.7
	M	– 27.4	– 26.1	116.6	36.2	– 7.5	– 15.3	70.0	38.0	96.8	43.7	29.0	18.8

#### Population data

In order to calculate the rate, population data by age, sex, province and year were obtained from the censuses conducted Iran every five years (https://www.amar.org.ir), specifically in 1996, 2006, 2011, and 2016. A growth model and annually population estimates were also used to project the population forward to 2030, covering the 5-year period.

#### Covariates variable

The Households Income and Expenditure Survey (HIES) was started in 1963 in Iranian rural areas. The HIES survey has been conducted Iranian urban areas since 1968 to assess the socioeconomic situation and estimating the average income and household expenses. Data for the wealth index, years of schooling, and urbanization until 2015 by age, sex, and province were derived from this survey^17^. Additionally, wealth index, years of schooling, and urbanization covariates values until 2030 were estimated using the spline method and then incorporated into the covariate model.

#### Projection

A spatio-temporal model was used to project the prevalence of diabetes, smoking, and PI. Spatio-temporal modeling refers to analyzing data with variations in both space and time. This type of model accounts for both spatial and temporal dependencies, with the specificmodel used here being a space–time autoregressive model. The model included a spatial correlation structure based on distance, meaning that closer locations have a stronger influence on each other's predicted values.

The dependent variable in the BMA was the prevalence of hypertension, overweight and obesity, and average salt intake, with time as the independent variable. Therefore, Bayesian model averaging (BMA) was chosen as a statistical method to incorporates model uncertainty by averaging over a set of models, each weighted by its posterior probability. This addresses model selection uncertainty, especially when dealing with many potential predictor variables, leading to more robust and reliable estimates. Wealth index, years of schooling, urbanization and population were entered as covariates. The projection was carried out from 2016 to 2030 at national and subnational levels. All analysis and figures/maps were drawn using R software version 4.3.2 (2023-10-31 ucrt).

## Results

### Trend and projection of non-communicable diseases risk factors

Table 2 and Appendix 1 (Tables 3–8) present the estimated changes in NCD risk factors in Iran from 2001 to 2030. This includes data observed in 2001 and 2016, along with projections for 2030 generated by both spatiotemporal and BMA models. The tables cover both national and subnational levels and present data for both men and women.

### Smoking

**Figure 1:**
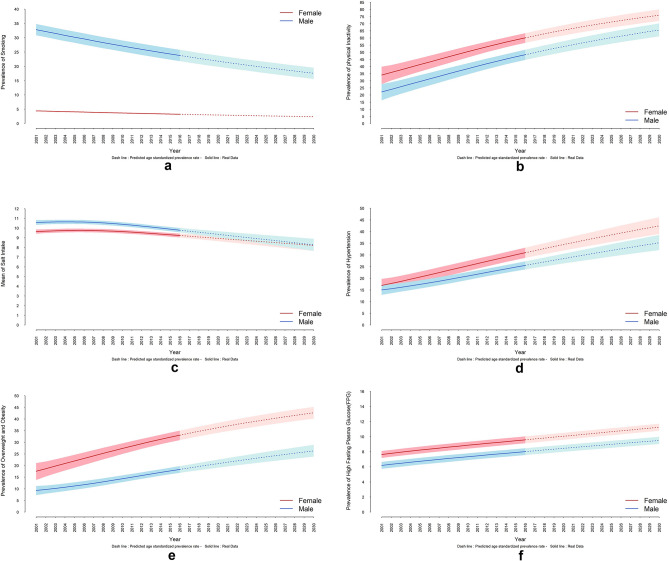
Trend and projection of age standardized prevalence of risk factors by sex from 2001 to 2030. (**a**) smoking, (**b**) physical inactivity, (**c**) mean of salt intake, (**d**) hypertension, (**e**) overweight and obesity, (**f**) Diabetes (created using R software version 4.3.2).

Between 2001 and 2016, the age-standardized prevalence of smoking in Iran has steadily decreased by nearly 27% for both men and women (Table 2). This decrease is projected to continue, with the prevalence of smoking for women and men projected to reach 2.4% and 17.6%, respectively, by 2030 (Appendix 1 & Fig. 1a).

**Figure 2:**
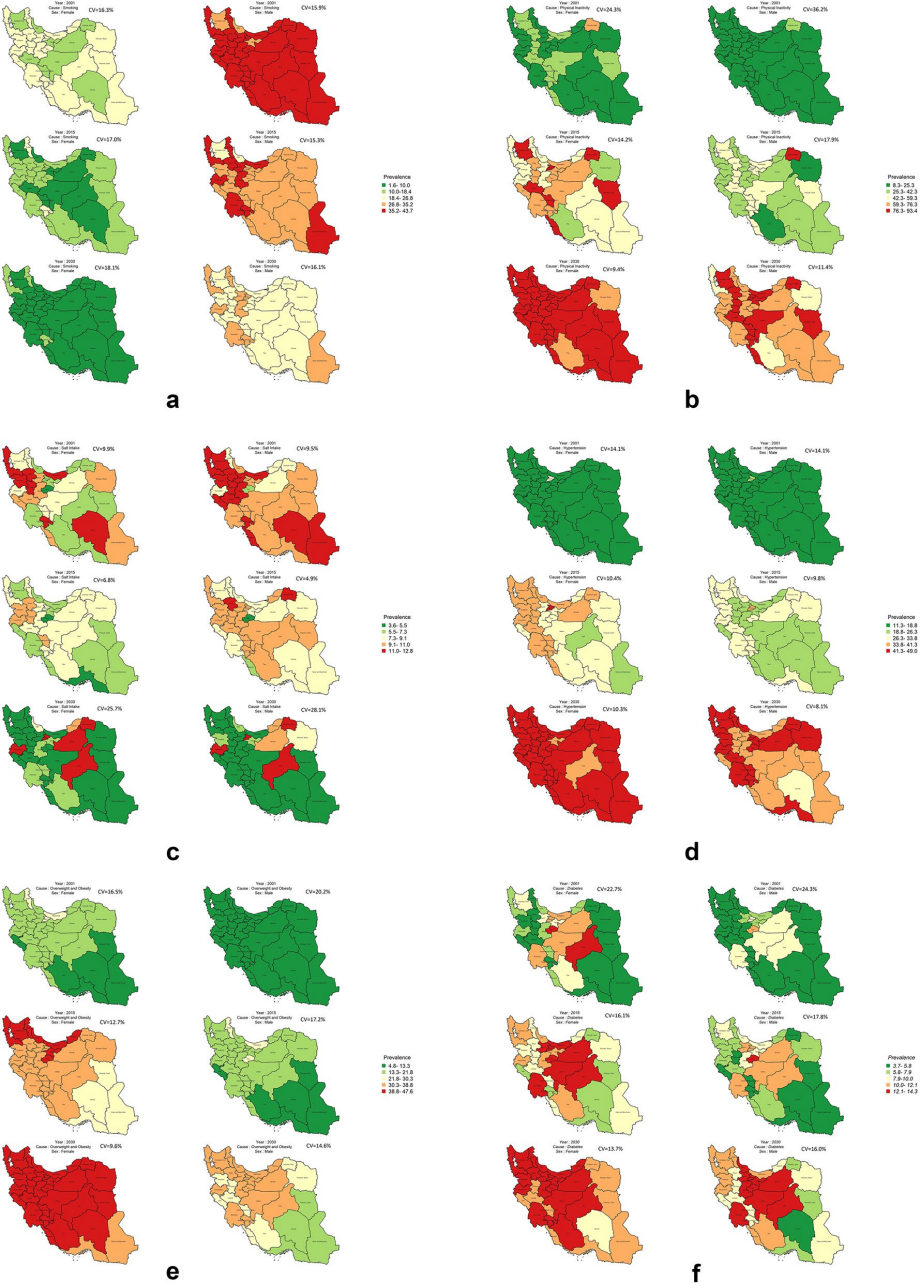
Distribution of age standardized prevalence of risk factors at subnational level in 2001, 2015, and 2030 by sex. (**a**) smoking, (**b**) physical inactivity, (**c**) mean of salt intake, (**d**) hypertension, (**e**) overweight and obesity, (**f**) diabetes (created using R software version 4.3.2).

The 2016 survey revealed differences in smoking prevalence across geographic regions for both genders, with the largest disparity observed among women (a 17% difference). These disparities are projected to widen by 2030, reaching an 18.1% difference (Fig. 2a).

### Physical inactivity

Between 2001 and 2016, the age-standardized prevalence of physical inactivity (PI) in Iran steadily increased (Fig. 1b). This increase represents a percent change of 75.2% for women and 116.6% for men (Table 2). Projections suggest that the PI prevalence is going to further increase, reaching 36.2% for men and 26.7% for women in 2030. These trends are illustrated in detail in Table 4 of the appendix, which shows estimates of age-standardized prevalence of PI by gender and province for 2001, 2016, and projections to 2030. Notably, the table reveals that the prevalence is projected to keep increasing, reaching 64% for men and 74.4% for women by 2030. Furthermore, geographic disparities in PI prevalence were observed among provinces for both genders. It is projected that this inequality will decrease in both genders in 2030 (9.4% in women and 11.4% in men) (Fig. 2b).

### Mean of salt intake

Between 2001 and 2016, there was a decrease in mean salt intake of 4.2% for women and 7.5% for men (Table 2). However, projections suggest a reversal of this trend, with both genders expected to have a mean salt intake of nearly 8 g per day in 2030, representing increases of 10.9% for women and 15.3% for men (Table 2 & Fig. 1c). While geographic disparities in mean salt intake were observed, projections indicate a significant widening of these disparities by 2030, with a three-fold increase for women and a five-fold increase for men among different provinces (Fig. 2c).

### Hypertension

Between 2001 and 2016, hypertension prevalence steadily increased, with substantial percent changes in age-standardized prevalence for both genders (82.8% for women and 70.0% for men) (Table 2 & Fig. 1d). While a further increase of nearly 30.0% is predicted for both genders by 2030 (Table 2 & Fig. 1d), projections suggest a potential slowdown in the rate of increase. Projections indicate a continued rise in prevalence, but at a slower pace, reaching an estimated age-standardized prevalence of 42.5% for women and 35.2% for men by 2030 (Appendix, Table 6). While geographic disparities in hypertension prevalence existed in 2001 (a 14.1% difference across provinces for both genders), these disparities are projected to decrease by 2030 (Fig. 2d).

### Obesity and overweight

Figure 1e shows a significant increase in the age-standardized prevalence of obesity and overweight for both genders between 2001 to 2016, with a substantial upward shift projected for 2030. Table 2 details the age-standardized prevalence of obesity and overweight by gender and provinces for 2001, 2016, and projections for 2030. A substantial increase is projected for 2030, resulting in an obesity and overweight prevalence of 42.6% for women and 26.3% for men (Appendix, Table 7). The widest disparities of age-standardized prevalence of obesity and overweight was observed in women in 2001 (percent change: 20.2%). The projection also shows a reduction in this variation by 2030 in both genders (Fig. 2e).

### Diabetes

Between 2001 and 2016, the age-standardized prevalence of diabetes increased by nearly 29.0% in men and 26.3% increase in women (Table 2). Figure 1f and Table 8 in the appendix show the trend for the age-standardized prevalence of diabetes for 2016. The projection indicates a gradual rise continuing until 2030, resulting in an age-standardized prevalence of 11.2% for women and 9.5% for men (Appendix, Table 8). The age-standardized mortality rate for diabetes in different provinces was higher in men than in women, and these disparities are expected to decrease by 2030 (Fig. 2f).

## Discussion

In this comprehensive study analyzed trends in the prevalence of significant major NCD risk factors including smoking, salt consumption, PI, overweight and obesity, high blood pressure, and diabetes at the national and sub-national levels from 2001 to 2016 with projections to 2030. The findings revealed positive trends for smoking prevalence and mean salt intake (decreases), but concerning trends for diabetes, hypertension, obesity, and physical inactivity (increases) from 2001 to 2016. These increases are projected to continue until 2030.

### Smoking

This study observed a decreasing trend in smoking prevalence, with a moderate slope in men and a slight slope in women. Notably, the prevalence decrease was similar in both genders. In recent years, studies in Iran, such as the one by Ghelichkhani et al. have demonstrated a decrease in smoking prevalence for both genders from 1990 to 2016^18^. Similar decreasing trends in smoking prevalence have been observed in many countries. A systematic analysis study covering 195 countries between 1990 and 2015 revealed a 28.4% decrease in smoking among men and a 34.4% decrease among women. This study indicated that a significant number of countries experienced a notable decline in annual smoking prevalence between 1990 and 2015^19^.

The implementation of the WHO Framework Convention on Tobacco Control (FCTC)^20^, as seen in policies adopted by Pakistan, Panama, and India in the last decade, has significantly accelerated the decline in smoking prevalence. These countries have experienced a more substantial drop in smoking prevalence since 2005 compared to the earlier period (1990–2005)^21^.

Interestingly, several countries, including Australia, Brazil, Canada, South Korea, and the United States achieved significant reductions in smoking prevalence even before the adoption of the FCTC^22–24^. These achievements were primarily driven by implementation of comprehensive tobacco control policies, such asadvertising restrictions, smoking bans in public places, and increased tobacco taxes^25–28^.

Although smoking rates in Iran have slightly decreased in recent years, attributing this decline solely to the implementation of tobacco control policies is challenging. This difficulty stems from the inadequacy of current policy execution. Evidence suggests the economic recession in Iran in recent years has led to a decreased cigarette consumption due to affordability concerns, resulting in a towards purchasing single-stick cigarettes instead of whole boxes^29,30^.

Controlling smoking in populations yet to experience widespread tobacco-related health issues, specifically preventing children, teenagers, and young adults from initiating smoking, is crucial in this arena^19^. Implementation and strengthening these policies in Iran could accelerate the downward trend projected in this study for the year 2030.

### Physical inactivity

In this study, among other known risk factors, PI emerged the most prevalent risk factor and is projected to continue rising until 2030. These findings show that although the prevalence of PI in women is higher than in men and is expected to persist until 2030, the rate of PI increase is steeper for men.

This study aligns with global findings of high and rising PI prevalence. A Saudi Arabian study by Al-Nozha et al. found an alarming 96.1% PI prevalence in 30 to 70 year-old adults (98.1% women, 93.9% men) which is considered a statistically significant difference^31^. Similarly, data from the US Behavioral Risk Factor Surveillance System (BRFSS) showed that 73.6% lacking sufficient PI^32^. High physical inactivity (PI) is not unique to Iran. Other studies report similarly high PI prevalence: Greece (69% men, 73% women)^33^, and Portugal (79% men, 86% women)^34^. This concern is further emphasized by the World Health Organization's (WHO) 2008 estimate of global PI prevalence for people aged 15 and older at 31% (28% men and 34% women), highlighting its public health significance. Factors driving this growing public health issue include reliance on cars, lack of parks and walkways, limited sports facilities, air pollution, elevator/escalator use, excessive screen time, and more. PI, a complex multi-dimensional issue, influenced by intra-personal, cultural, social, economic, and environmental factors, not only significantly increases healthcare costs but also hinders other healthy behaviors such as healthy eating and quitting smoking. Therefore, addressing PI effectively holds considerable power in controlling NCDs.

### Salt intake

Our study observed a decreasing in salt intake among both men and women in Iran from 2001 to 2016, and this decline is projected to continue until 2030. According to the findings, salt intake was higher in men than in women. While men initially had higher salt intake, the gender gap narrowed from 2015 onwards and is projected to completely disappear by 2030, with both genders reaching an average intake of around 8.2 g per day.

In many parts of the world, including Iran where daily salt intake ranges from 8 to 12 g per day, consumption significantly surpasses the World Health Organization's recommended limit of 5 g per day^35,36^. While the current study projects a decline to an average of 8.2 g per day by 2030, this figure still significantly surpasses the necessary amount. Therefore, more aggressive intervention strategies are imperative to reduce salt consumption to the WHO’s recommended levels by 2030.

Reducing salt intake can be a very effective strategy to deal with the cardiovascular disease epidemic^37–39^. Despite compelling evidence linking reduced salt intake to a decline in cardiovascular disease, few measures have been taken in developing countries. While initiatives exist in countries such as Iran, China, Pakistan, Bangladesh, Nepal, Cuba, Kenya, and Ghana, many developing countries still lack comprehensive dietary guidelines and salt reduction programs^37^. However, global efforts are gaining momentum, particularly in developed nations to reduce salt intake. In a systematic review by Trieu et al. the results showed that currently a total of 75 countries have adopted national salt reduction strategies. These strategies are often multi-faceted, encompassing industry engagement for product formulations (61 countries), sodium content targets (39 countries), consumer education (71 countries), food labeling (31 countries), targeted taxation (3 countries), and interventions within government and public institutions (54 countries). Notably, 33 of these implementing countries fall under the low- and middle-income category^38^.

The United Kingdom’s salt reduction strategy is exemplified as an effective model in combatting dietary sodium excess on a national scale, meriting consideration by other countries. The United Kingdom's gradual salt reduction strategy in processed foods, implemented without impacting consumer awareness^40^. In contrast, Iran prioritizes active policy interventions. The effective strategies used in Iran to reduce salt intake include reducing salt in processed foods, implementing food labeling, educating the public, supporting relevant controlling departments, and implementing a national salt reduction campaign^36^.

### Hypertension

This study projects a rising prevalence of hypertension, with women currently exhibiting higher rates than men. The prevalence of hypertension in adults has been widely studied. Although the results depended on the definition of hypertension, in most of the studies show increasing hypertension prevalence in developing countries and stable trend in developed countries. For example, China saw a rise from 23.8% in 1991 to 31.5% in 2009^41^, and West Africa experienced a jump from 12.9% in 1980 to 34.4% in 2014^42^. In contrast, developed countries like Canada and the United States have maintained relatively stable prevalence^43,44^. This disparity likely stems from differing approaches toward hypertension. High-income countries often implement comprehensive programs targeting risk factors like salt intake, smoking, and physical inactivity, contributing to their success in controlling hypertension prevalence. Developed countries may have achieved a stable hypertension prevalence because they experienced its rise earlier and implemented preventive measures alongside industrialization, which helped to curb the trend. In contrast, developing countries grapple with rising prevalence driven by factors like processed food consumption, physical inactivity, growing industrialization, and increasing obesity. Notably, this study aligns with research from Germany, where similarly defined hypertension showed higher prevalence in women across age groups. However, the gender gap narrows with age, with both genders exhibiting almost similar prevalence in the 65–84 age group^45,46^. Similar trends observed in other studies suggest sex hormones may play a role in the higher prevalence of hypertension^47–49^. Moreover, researches have shown that the prevalence of hypertension increases with age; for instance, the National Health and Nutrition Examination Survey (NHANES) data indicates hypertension prevalence to rise from 7.3% in in the 18 to 39-year age bracket to 66.3% in people over 60 age remains a key contributor, but unhealthy lifestyle choices like alcohol and tobacco use, physical inactivity, and increasing BMI over time likely further exacerbate the uptrend^49–52^.

### Overweight and obesity

This study projects a concerning rise in overweight and obesity in Iran, with the trend continuing until 2030. The findings showed that the prevalence of overweight and obesity was higher in women than in men, but the rate of increase from 2001 to 2030 in men was higher than in women.

In other studies, the increasing trend of overweight and obesity in the world has been shown. The results of a comprehensive review study, based on 1698 population-based studies with 19.2 million participants (9.9 million men and 9.3 million women) in 200 countries, showed that the age-standardized prevalence of obesity increased from 3.2 to 10.8% in men and from 6.4 to 14.9% in women between 1975 and 2014^53^. During the last four decades, the world has witnessed a transition from underweight to obesity, so that the prevalence of obesity has doubled. People in the world are more obese than underweight, and this situation is in all regions of the world, except for sub-Saharan Africa and South Asia^53^. The increase in BMI since 2000 compared to previous decades has decreased in high-income countries, while obesity has become a major public health concern in most middle-income countries during this time. In general, due to the rapid increase in BMI in most regions of the world, it is hard to conclude that the global increase in BMI has slowed down^54,55^.

According to the findings, the prevalence of obesity in women was significantly higher than in men, which was consistent with the studies conducted in this field. Studies have shown that women are more at risk of being overweight and obese than men, which can have extensive effects on reproductive health and especially on their pregnancy since obese women are at higher risk of gestational diabetes, preeclampsia, premature birth, fetal macrosomia, and newborn diseases. The cause of obesity is very complex and includes a set of genetic, environmental, physiological, cultural, political, social, and economic factors that make effective interventions in this field challenging. Among the most important reasons for the increase in BMI and the prevalence of obesity in women compared to men are a lower percentage of muscle tissue and higher fat in women, female hormones during puberty, use of contraceptives, weight gain due to pregnancy, hormonal disorders due to menopause, and inactivity^56–58^. Therefore, it is necessary to pay attention to these factors in intervention programs to reduce the prevalence of obesity in women.

### Diabetes

The present study showed that the prevalence of diabetes in the age group of 30–70 years in both genders has increased from 2001 to 2016, with projections indicating a further increase by 2030. The prevalence of diabetes in women has increased from 7.6% in 2001 to 9.6% in 2016, with an estimated increase to 11.2% in 2030. Similarly, in men, the prevalence demonstrated a growing trend, increasing from 6.2% in 2001 to 8.0% in 2016, and is projected to reach 9.5% by 2030. The overall projected increase during the 30 years of the study was 47.4% for women and 53.2% for men.

Other studies have also confirmed the increased prevalence of diabetes. Ezzati et al. showed that the age-standardized prevalence of diabetes in men increased from 4.3 to 9.0% and from 5.0 to 7.9% in women between 1980 and 2014. They concluded that the age-standardized prevalence of diabetes has increased since 1980 in all countries or, at best, has remained unchanged. Along with population growth and aging, this increase has contributed to a fourfold rise in the number of adults with diabetes worldwide. Furthermore, the burden of diabetes, in terms of both prevalence and the number of affected adults, risen more rapidly in low- and middle-income countries than in high-income countries^59^. In another study, which was conducted to estimate the global prevalence of diabetes in 2013 and project it through to 2035 found that the number of people with diabetes increased from 382 million in 2013 to an expected 592 million in 2035. The study highlighted that the majority of individuals with diabetes reside in low- and middle-income countries, which are predicted to see the sharpest rise in diabetes prevalence over the next 22 years^60^.

Studies have shown that diabetes is becoming more prevalent in most parts of the world. Lin et al. conducted a study using data from 195 countries and found that the incidence and prevalence of diabetes increased from 1990 to 2017 worldwide. It is predicted that this trend will continue until 2025^61^. In the United States, a study on trends in diabetes prevalence in adults revealed that the prevalence of diabetes has been increasing from 1999 to 2018^62^. However, a review study suggests that the incidence of type 2 diabetes increased from 1990 to 2005, but then slowed down significantly from 2006 to 2014. It is important to note that a limitation of this study was that it mostly included data from high-income countries, while diabetes trends may differ in low- and middle-income countries^63^. Overall, the reasons for the increase in the prevalence of diabetes can be attributed to screening and early diagnosis of patients, treatment of patients, increase in life expectancy, a decline in mortality from to infectious diseases, aging populations, changes in lifestyle and diet, urbanization, and decreased PI.

### Strengths and limitations

This study benefits from utilizing data collected over several years from a diverse population across all Iranian provinces. This national scope and the population's heterogeneity enhance the generalizability of our findings to the entire Iranian population. Furthermore, the study's strength lies in its simultaneous measurement of six major NCDs risk factors, making it a valuable contribution to research in this area, particularly for other middle-income countries within the Eastern Mediterranean Region (EMRO).

One limitation of this study is the inherent uncertainty of projections, a common issue in all projection-based studies. Unpredictable factors such as the introduction of new, effective preventive and therapeutic measures, advances in technology and nanomedicine in disease’s treatment, or macro-economic shocks can influence the projected trends and cause deviations. The covid-19 pandemic was a stark example of such unpredictable factors. Additionally, data on alcohol use was not available in the country; consequently, this risk factor could not be included in the study, which represents another limitation. One important limitation we faced was the unavailability of data from the most recent STEPs survey. Since the complete STEPs survey data has not yet been publicly published in details for reserarchers. Given the limited amount of published data available after 2016 and GBD 2019, it is highly unlikely that incorporating the latest data would meaningfully alter the projections.

## Conclusion

This study showed that the prevalence of four main risk factors— PI, overweight and obesity, hypertension, and diabetes—is increasing in Iran. Although the prevalence of the two risk factors of smoking and salt intake is decreasing in Iran, this reduction is insufficient and the levels still remain higher than the recommended. Considering the inconvenient health associated with the studied risk factors and their impact on NCDs, it is essential to implement effective interventions that encourage the adoption of a healthy lifestyle and reduction of the risk factors. Such interventions could include the design and creation of an online system for registering individual risk factors, which would be managed at the level of the Ministry of Health. Additionally, the integration of cost-effective strategies to reduce NCD risk factors into the primary health care system at all levels could increase universal coverage.

### Suggestions for future research and policy implications

Diverse population-based studies that examine the interaction between various risk factors—such as poor diet, PI, tobacco use, and harmful use of alcohol—and the onset of NCDs will be instrumental in formulating targeted interventions. Also, the cost-effectiveness of interventions for reducing risk factors should also be considered. Future research and policy-making need to go hand in hand to create comprehensive strategies that address the complexity of NCD risk factors. Only with a multidisciplinary approach that includes robust research, informed policy, innovation, and global cooperation can we hope to significantly reduce the impact of NCDs on global health.

### Supplementary Information

Supplementary Tables.

## Data Availability

The datasets generated and/or analysed during the current study are not publicly available. They are maintained by Ministry of Health and Medical Education (MOHME) of Iran, but can be accessed through the corresponding author on reasonable request.
