# Supplementary material for: Trend and projection of non-communicable diseases risk factors in Iran from 2001 to 2030
Source: Sci Rep. 2024 Apr 6;14:8092. doi: 10.1038/s41598-024-58629-z (PMC10998837; doi:10.1038/s41598-024-58629-z)
Supplement: Supplementary file 1 — Supplementary Tables. [file 41598_2024_58629_MOESM1_ESM.docx]

Appendix 1

| Table 1. additional data sources used to estimate the prevalence and trends of diabetes, hypertension and obesity | | | |
| --- | --- | --- | --- |
| # | **Data source** | **Year used** | **Level** |
| **1** | **Golestan Cohort Study (GCS)** | **2006** | **Subnational** |
| **2** | **The Isfahan Healthy Heart Program (IHHP)** | **2001**  **2007** | **Community** |
| **3** | **MONICA project in Iran** | **2004** | **Subnational** |
| **4** | **National Health Survey** | **2000** | **National** |
| **5** | **Tehran Lipid and Glucose Study (TLGS)** | **2001**  **2005**  **2008**  **2011** | **Community** |
| **6** | **Systematic Review** | **2015-1994** | **Subnational and Community** |
| **7** | **burden and risk factors of non-communicable diseases in Fars province (Pars Cohort)** | **2013** | **Subnational** |

Table 2. Steps surveys sample size

| Survey number | Year | Sample size |
| --- | --- | --- |
| 1 | 2005 | 80032 |
| 2 | 2006 | 29976 |
| 3a | 2007 | 30000 |
| 3b | 2007 | 5000 |
| 4 | 2008 | 29938 |
| 5 | 2009 | 39996 |
| 6 | 2011 | 12459 |
| 7 | 2016 | 30541 |

| Table 3. Age standardized prevalence of smoking at three time periods in national and subnational levels | | | | | | | | | | | | |
| --- | --- | --- | --- | --- | --- | --- | --- | --- | --- | --- | --- | --- |
| province | 2001 | | | | 2016 | | | | 2030 | | | |
|  | Female | | Male | | Female | | Male | | Female | | Male | |
|  | P | 95%CI | P | 95%CI | P | 95%CI | P | 95%CI | P | 95%CI | P | 95%CI |
| Markazi | 5.5 | (5.4- 5.6) | 39.8 | (37.8- 41.7) | 3.9 | (3.8- 4.0) | 29.8 | (27.8- 31.8) | 2.8 | (2.7- 2.9) | 22.6 | (20.5- 24.6) |
| Gilan | 3.3 | (3.2- 3.4) | 24.5 | (22.7- 26.4) | 2.3 | (2.2- 2.4) | 17.7 | (15.6- 19.6) | 1.6 | (1.5- 1.7) | 13.2 | (11.1- 15.2) |
| Mazandaran | 5.1 | (5.0- 5.2) | 37.0 | (35.1- 39.0) | 3.5 | (3.4- 3.6) | 26.1 | (24.2- 28.1) | 2.5 | (2.4- 2.6) | 18.8 | (16.7- 20.8) |
| Azerbaijan_East | 3.1 | (3.0- 3.2) | 22.7 | (20.8- 24.8) | 2.3 | (2.2- 2.4) | 16.6 | (14.7- 18.6) | 1.7 | (1.6- 1.8) | 12.2 | (10.1- 14.1) |
| Azerbaijan_West | 5.1 | (5.0- 5.2) | 38.4 | (36.4- 40.5) | 3.9 | (3.8- 4.0) | 28.3 | (26.4- 30.4) | 3.0 | (2.9- 3.1) | 21.1 | (19.1- 23.0) |
| Kermanshah | 4.9 | (4.8- 5.0) | 36.7 | (34.8- 38.6) | 3.8 | (3.7- 3.9) | 26.9 | (25.1- 28.8) | 2.9 | (2.8- 3.0) | 20.3 | (18.3- 22.1) |
| Khuzestan | 5.2 | (5.1- 5.3) | 39.3 | (37.4- 41.3) | 3.9 | (3.8- 4.0) | 28.5 | (26.7- 30.4) | 2.9 | (2.8- 3.0) | 20.4 | (18.6- 22.4) |
| Fars | 4.6 | (4.5- 4.7) | 34.1 | (32.3- 36.1) | 3.4 | (3.3- 3.5) | 24.6 | (22.7- 26.7) | 2.5 | (2.4- 2.6) | 17.9 | (15.9- 19.9) |
| Kerman | 3.5 | (3.4- 3.6) | 26.8 | (24.9- 28.8) | 2.6 | (2.5- 2.7) | 20.1 | (18.3- 22.1) | 1.9 | (1.8- 2.0) | 15.4 | (13.4- 17.2) |
| Khorasan_Razavi | 4.2 | (4.1- 4.3) | 31.6 | (29.6- 33.5) | 3.1 | (3.0- 3.2) | 23.0 | (21.0- 25.0) | 2.4 | (2.3- 2.5) | 17.1 | (15.0- 19.1) |
| Isfahan | 3.7 | (3.6- 3.8) | 27.6 | (25.6- 29.6) | 2.9 | (2.8- 3.0) | 21.6 | (19.7- 23.5) | 2.3 | (2.2- 2.4) | 17.3 | (15.2- 19.2) |
| Sistan&Baluchistan | 4.6 | (4.5- 4.7) | 34.8 | (32.7- 36.8) | 3.6 | (3.5- 3.7) | 26.0 | (24.1- 28.0) | 2.8 | (2.7- 2.9) | 19.8 | (17.9- 21.7) |
| Kurdistan | 4.3 | (4.2- 4.4) | 32.3 | (30.4- 34.4) | 3.3 | (3.2- 3.4) | 23.0 | (20.9- 24.9) | 2.6 | (2.5- 2.7) | 16.4 | (14.4- 18.2) |
| Hamadan | 4.4 | (4.3- 4.5) | 32.7 | (30.7- 34.7) | 3.2 | (3.1- 3.3) | 23.3 | (21.3- 25.5) | 2.3 | (2.2- 2.4) | 16.7 | (14.7- 18.7) |
| ChaharM&Bakhtiari | 4.0 | (3.9- 4.1) | 29.1 | (27.2- 31.0) | 2.9 | (2.8- 3.0) | 21.4 | (19.4- 23.2) | 2.1 | (2.0- 2.2) | 15.9 | (13.9- 17.9) |
| Lorestan | 4.0 | (3.9- 4.1) | 29.9 | (27.8- 31.9) | 2.9 | (2.8- 3.0) | 22.2 | (20.4- 24.2) | 2.2 | (2.1- 2.3) | 16.8 | (14.7- 18.8) |
| Ilam | 4.3 | (4.2- 4.4) | 31.3 | (29.4- 33.3) | 3.1 | (3.0- 3.2) | 22.2 | (20.2- 24.2) | 2.3 | (2.2- 2.4) | 15.9 | (14.0- 18.0) |
| Kohgiluyeh&BoyerA | 5.9 | (5.8- 6.0) | 42.7 | (40.6- 44.6) | 4.3 | (4.2- 4.4) | 29.8 | (27.9- 31.8) | 3.3 | (3.2- 3.4) | 21.5 | (19.6- 23.4) |
| Bushehr | 4.5 | (4.4- 4.6) | 32.7 | (30.9- 34.7) | 3.2 | (3.1- 3.3) | 23.3 | (21.2- 25.2) | 2.3 | (2.2- 2.4) | 16.8 | (14.8- 18.8) |
| Zanjan | 4.7 | (4.6- 4.8) | 35.2 | (33.3- 37.2) | 3.5 | (3.4- 3.6) | 25.7 | (23.9- 27.6) | 2.5 | (2.4- 2.6) | 18.6 | (16.7- 20.5) |
| Semnan | 3.5 | (3.4- 3.6) | 25.8 | (23.8- 27.8) | 2.4 | (2.3- 2.5) | 18.8 | (16.8- 20.8) | 1.7 | (1.6- 1.8) | 14.2 | (12.2- 15.9) |
| Yazd | 4.1 | (4.0- 4.2) | 30.0 | (28.0- 31.9) | 2.9 | (2.8- 3.0) | 20.9 | (18.9- 23.0) | 2.0 | (1.9- 2.1) | 14.5 | (12.4- 16.4) |
| Hormozgan | 4.2 | (4.1- 4.3) | 31.8 | (29.9- 33.6) | 3.0 | (2.9- 3.1) | 22.8 | (20.8- 24.6) | 2.2 | (2.1- 2.3) | 16.8 | (14.8- 18.8) |
| Tehran | 3.3 | (3.2- 3.4) | 24.9 | (23.0- 26.9) | 2.5 | (2.4- 2.6) | 19.9 | (18.0- 21.9) | 2.0 | (1.9- 2.1) | 16.5 | (14.4- 18.5) |
| Ardabil | 6.0 | (5.9- 6.1) | 43.7 | (41.7- 45.6) | 4.4 | (4.2- 4.4) | 30.7 | (28.7- 32.7) | 3.2 | (3.1- 3.3) | 21.9 | (19.9- 23.8) |
| Qom | 4.3 | (4.2- 4.4) | 31.1 | (29.1- 32.9) | 3.2 | (3.1- 3.3) | 23.6 | (21.6- 25.5) | 2.3 | (2.2- 2.4) | 17.5 | (15.5- 19.4) |
| Qazvin | 5.2 | (5.1- 5.3) | 39.0 | (37.0- 41.0) | 3.9 | (3.8- 4.0) | 30.2 | (28.2- 32.2) | 3.0 | (2.9- 3.1) | 24.3 | (22.4- 26.2) |
| Golestan | 4.9 | (4.8- 5.0) | 35.5 | (33.6- 37.4) | 3.5 | (3.4- 3.6) | 24.7 | (22.6- 26.8) | 2.6 | (2.5- 2.7) | 17.5 | (15.5- 19.4) |
| Khorasan_North | 4.0 | (3.9- 4.1) | 30.6 | (28.4- 32.5) | 2.9 | (2.8- 3.0) | 20.8 | (19.0- 22.7) | 2.1 | (2.0- 2.2) | 14.4 | (12.5- 16.4) |
| Khorasan_South | 4.4 | (4.3- 4.5) | 32.9 | (31.1- 34.9) | 3.1 | (3.0- 3.2) | 21.9 | (20.0- 23.9) | 2.2 | (2.1- 2.3) | 15.1 | (13.1- 16.9) |
| Alborz | 4.2 | (4.1- 4.3) | 31.7 | (29.6- 33.6) | 3.1 | (3.0- 3.2) | 24.4 | (22.5- 26.3) | 2.3 | (2.2- 2.4) | 19.2 | (17.2- 21.1) |
| **Iran** | **4.4** | **(4.3- 4.5)** | **32.8** | **(30.9- 34.8)** | **3.2** | **(3.1- 3.3)** | **23.8** | **(21.8- 25.8)** | **2.4** | **(2.3- 2.5)** | **17.6** | **(15.6- 19.5)** |

| Table 4. Age standardized prevalence of physical inactivity at three time periods in national and subnational levels | | | | | | | | | | | | | |
| --- | --- | --- | --- | --- | --- | --- | --- | --- | --- | --- | --- | --- | --- |
| province | 2001 | | | | 2016 | | | | 2030 | | | | |
|  | Female | | Male | | Female | | Male | | Female | | Male | | |
|  | P | 95%CI | P | 95%CI | P | 95%CI | P | 95%CI | P | 95%CI | P | 95%CI |  |
| Markazi | 29.7 | (24.0- 35.1) | 17.7 | (12.3- 22.8) | 54.8 | (51.3- 58.0) | 43.0 | (39.4- 46.5) | 71.0 | (66.8- 75.4) | 60.5 | (55.8- 65.5) |  |
| Gilan | 30.2 | (24.4- 35.6) | 18.3 | (12.9- 23.4) | 55.4 | (51.9- 58.6) | 43.6 | (40.0- 47.0) | 71.4 | (67.5- 75.5) | 60.9 | (56.3- 65.7) |  |
| Mazandaran | 36.2 | (30.2- 41.9) | 24.4 | (18.7- 29.7) | 61.6 | (58.1- 64.7) | 49.8 | (46.2- 53.1) | 76.9 | (73.2- 80.7) | 66.6 | (62.3- 71.0) |  |
| Azerbaijan_East | 43.6 | (37.2- 49.5) | 31.7 | (25.9- 37.2) | 68.8 | (65.5- 71.9) | 57.0 | (53.5- 60.3) | 83.2 | (79.5- 86.9) | 73.0 | (69.0- 77.2) |  |
| Azerbaijan_West | 24.5 | (18.8- 29.9) | 12.7 | (7.3- 17.8) | 49.8 | (46.3- 53.2) | 37.9 | (34.3- 41.4) | 66.7 | (62.3- 71.4) | 55.6 | (51.1- 60.2) |  |
| Kermanshah | 28.6 | (22.8- 34.1) | 16.6 | (11.1- 21.9) | 53.7 | (50.2- 57) | 42.0 | (38.4- 45.4) | 70.0 | (65.9- 74.1) | 59.4 | (55.0- 64.0) |  |
| Khuzestan | 32.1 | (26.2- 37.5) | 20.1 | (14.6- 25.3) | 57.3 | (53.8- 60.5) | 45.6 | (42.0- 49.0) | 73.1 | (69.2- 77.2) | 63.1 | (58.1- 68.8) |  |
| Fars | 15.9 | (10.5- 21.1) | 8.3 | (3.0- 13.3) | 41.4 | (37.7- 44.8) | 29.8 | (26.1- 33.2) | 58.8 | (53.7- 64.1) | 48.6 | (42.7- 54.5) |  |
| Kerman | 26.0 | (20.2- 31.4) | 14.1 | (8.6- 19.4) | 51.4 | (47.9- 54.7) | 39.5 | (35.9- 43.0) | 68.0 | (63.9- 72.3) | 57.1 | (52.8- 61.6) |  |
| Khorasan_Razavi | 22.8 | (17.2- 28.1) | 10.9 | (5.6- 16.0) | 48.0 | (44.4- 51.3) | 36.2 | (32.6- 39.7) | 64.9 | (60.5- 69.4) | 54.1 | (49.5- 58.9) |  |
| Isfahan | 40.4 | (34.2- 46.2) | 28.5 | (22.7- 34.0) | 65.5 | (62.1- 68.6) | 53.8 | (50.3- 57.0) | 80.3 | (76.6- 83.9) | 70.1 | (66.0- 74.2) |  |
| Sistan&Baluchistan | 29.3 | (23.6- 34.8) | 17.4 | (11.9- 22.7) | 54.6 | (51.1- 57.9) | 42.8 | (39.2- 46.2) | 70.8 | (66.7- 74.9) | 60.0 | (55.8- 64.3) |  |
| Kurdistan | 25.8 | (20.1- 31.1) | 13.9 | (8.6- 19.0) | 51.0 | (47.5- 54.4) | 39.3 | (35.7- 42.7) | 67.7 | (63.5- 71.8) | 56.9 | (52.5- 61.4) |  |
| Hamadan | 39.7 | (33.5- 45.5) | 27.8 | (22.1- 33.3) | 64.9 | (61.5- 68.1) | 53.2 | (49.6- 56.5) | 80.0 | (76.2- 83.9) | 69.6 | (65.5- 74.0) |  |
| ChaharM&Bakhtiari | 41.6 | (35.3- 47.5) | 29.5 | (23.8- 35.0) | 66.9 | (63.5- 70.0) | 55.1 | (51.6- 58.4) | 81.6 | (78.0- 85.1) | 71.5 | (67.1- 76.2) |  |
| Lorestan | 41.5 | (35.0- 47.5) | 29.8 | (23.9- 35.3) | 66.8 | (63.5- 69.9) | 55.2 | (51.6- 58.4) | 81.2 | (77.7- 84.6) | 71.4 | (67.3- 75.6) |  |
| Ilam | 33.3 | (27.3- 38.9) | 21.5 | (15.9- 26.8) | 58.7 | (55.2- 61.9) | 46.9 | (43.3- 50.3) | 74.4 | (70.5- 78.4) | 63.9 | (59.4- 68.6) |  |
| Kohgiluyeh&BoyerA | 37.7 | (31.6- 43.3) | 25.7 | (20.1- 31.0) | 62.8 | (59.3- 65.9) | 51.0 | (47.5- 54.4) | 77.9 | (74.2- 81.7) | 67.7 | (63.4- 72.3) |  |
| Bushehr | 43.1 | (36.7- 49.0) | 31.2 | (25.4- 36.6) | 68.3 | (64.9- 71.4) | 56.5 | (53.0- 59.7) | 82.8 | (79.1- 86.5) | 72.6 | (68.4- 76.9) |  |
| Zanjan | 37.0 | (30.9- 42.7) | 25.1 | (19.5- 30.4) | 62.3 | (58.8- 65.5) | 50.4 | (46.9- 53.8) | 77.9 | (73.8- 82.0) | 67.2 | (62.8- 71.8) |  |
| Semnan | 33.8 | (27.9- 39.4) | 21.7 | (16.2- 26.9) | 59.0 | (55.5- 62.2) | 47.2 | (43.6- 50.6) | 74.8 | (70.8- 78.8) | 64.3 | (59.7- 69.3) |  |
| Yazd | 27.8 | (22.1- 33.3) | 15.9 | (10.4- 21.1) | 53.1 | (49.5- 56.4) | 41.3 | (37.7- 44.7) | 69.5 | (65.4- 73.8) | 58.9 | (54.3- 63.6) |  |
| Hormozgan | 24.8 | (19.2- 30.1) | 12.7 | (7.4- 17.8) | 50.0 | (46.5- 53.4) | 38.3 | (34.6- 41.7) | 66.8 | (62.5- 71.2) | 56.2 | (51.2- 61.5) |  |
| Tehran | 34.7 | (28.8- 40.2) | 23.0 | (17.5- 28.3) | 60.3 | (56.8- 63.5) | 48.4 | (44.8- 51.7) | 76.1 | (71.8- 80.9) | 65.3 | (60.9- 69.8) |  |
| Ardabil | 27.0 | (21.4- 32.4) | 15.2 | (9.8- 20.4) | 52.4 | (48.8- 55.7) | 40.5 | (36.9- 43.9) | 68.9 | (64.6- 73.4) | 58.0 | (53.5- 62.8) |  |
| Qom | 32.6 | (26.7- 38.2) | 20.8 | (15.2- 26.1) | 58.1 | (54.6- 61.3) | 46.3 | (42.7- 49.7) | 74.1 | (70.0- 78.5) | 63.5 | (59.0- 68.2) |  |
| Qazvin | 30.7 | (25.0- 36.2) | 18.9 | (13.4- 24) | 55.9 | (52.4- 59.2) | 44.2 | (40.6- 47.6) | 72.1 | (67.9- 76.4) | 61.5 | (56.9- 66.3) |  |
| Golestan | 27.3 | (21.6- 32.7) | 15.4 | (9.9- 20.7) | 52.7 | (49.2- 56.0) | 41.0 | (37.4- 44.5) | 69.3 | (65.0- 73.8) | 58.8 | (54.1- 63.6) |  |
| Khorasan_North | 56.4 | (48.6- 63.1) | 44.4 | (37.9- 50.4) | 81.9 | (78.8- 84.6) | 70.0 | (66.7- 73.1) | 93.4 | (90.5- 95.6) | 84.4 | (80.7- 87.9) |  |
| Khorasan_South | 41.7 | (35.3- 47.6) | 29.7 | (23.9- 35.2) | 66.8 | (63.5- 69.9) | 55.0 | (51.6- 58.3) | 81.4 | (77.9- 84.6) | 71.1 | (67.3- 74.7) |  |
| Alborz | 41.4 | (35.2- 47.2) | 29.6 | (23.9- 35.0) | 66.7 | (63.3- 69.8) | 54.8 | (51.3- 58.1) | 81.4 | (77.8- 84.9) | 71.1 | (66.9- 75.6) |  |
| **Iran** | **33.5** | **(27.5- 39.1)** | **21.7** | **(16.1- 27.0)** | **58.7** | **(55.3- 61.9)** | **47.0** | **(43.4- 50.3)** | **74.4** | **(70.4- 78.4)** | **64.0** | **(59.5- 68.6)** |  |

| Table 5. Age standardized prevalence of salt intake at three time periods in national and subnational levels | | | | | | | | | | | | | | |
| --- | --- | --- | --- | --- | --- | --- | --- | --- | --- | --- | --- | --- | --- | --- |
| province | 2001 | | | | 2016 | | | | | 2030 | | | | |
|  | Female | | Male | | Female | | Male | | | Female | | Male | | |
|  | P | 95%CI | P | 95%CI | P | 95%CI | P | 95%CI | P | | 95%CI | P | 95%CI |  |
| Markazi | 9.9 | (9.9- 10) | 11.9 | (11.6-12.3) | 9.7 | (9.7- 9.8) | 10.2 | (9.9- 10.5) | 9.2 | | (9.0- 9.4) | 8.0 | (7.2-8.8) |  |
| Gilan | 8.6 | (8.5- 8.7) | 10.3 | (10.2-10.4) | 9.1 | (9.0- 9.2) | 9.6 | (9.5- 9.7) | 9.6 | | (9.5- 9.8) | 8.5 | (8.2- 8.8) |  |
| Mazandaran | 10.7 | (10.3- 11.1) | 12.5 | (11.8-13.1) | 9.0 | (8.6- 9.3) | 9.5 | (8.9- 10.1) | 5.9 | | (5.0- 6.8) | 4.3 | (2.8- 5.8) |  |
| Azerbaijan_East | 9.5 | (9.3- 9.7) | 11.5 | (11-11.9) | 8.9 | (8.7- 9.1) | 9.6 | (9.2- 10.0) | 6.6 | | (5.9- 7.2) | 5.9 | (4.9- 6.9) |  |
| Azerbaijan_West | 11.7 | (11.2- 12.2) | 12.8 | (12.2-13.4) | 9.4 | (9.0- 9.9) | 9.8 | (9.3- 10.4) | 5.6 | | (4.3- 6.8) | 5.4 | (3.9- 6.9) |  |
| Kermanshah | 9.7 | (9.5- 9.8) | 9.6 | (9.5-9.8) | 10.5 | (10.4- 10.7) | 10.5 | (10.4- 10.7) | 11.2 | | (10.9- 11.5) | 11.2 | (10.9- 11.5) |  |
| Khuzestan | 9.2 | (9.1- 9.2) | 10.6 | (10.4-10.7) | 9.2 | (9.1- 9.2) | 9.7 | (9.6- 9.9) | 8.7 | | (8.6- 8.8) | 8.1 | (7.5- 8.7) |  |
| Fars | 9.0 | (9.0- 9.1) | 10.6 | (10.4-10.8) | 9.3 | (9.2- 9.4) | 9.9 | (9.7- 10.1) | 8.8 | | (8.6- 9.1) | 8.2 | (7.6- 8.7) |  |
| Kerman | 10.8 | (10.3- 11.3) | 12.0 | (11.4-12.6) | 8.8 | (8.3- 9.2) | 9.2 | (8.6- 9.8) | 4.5 | | (3.2- 5.7) | 3.7 | (2.2- 5.2) |  |
| Khorasan_Razavi | 10.2 | (10.1- 10.4) | 10.0 | (10.0-10.1) | 9.6 | (9.5- 9.8) | 9.7 | (9.6- 9.7) | 8.3 | | (7.9- 8.7) | 9.3 | (9.2- 9.5) |  |
| Isfahan | 9.5 | (9.4- 9.7) | 10.3 | (10.2-10.5) | 9.5 | (9.4- 9.6) | 10.1 | (9.9- 10.2) | 7.7 | | (7.2- 8.2) | 8.1 | (7.6- 8.6) |  |
| Sistan&Baluchistan | 10.0 | (9.8- 10.3) | 11.2 | (10.9-11.6) | 9.0 | (8.8- 9.2) | 9.7 | (9.4- 10.0) | 7.5 | | (7.1- 8.8) | 7.7 | (7- 8.3) |  |
| Kurdistan | 11.2 | (10.9- 11.5) | 10.7 | (10.5-10.9) | 10.2 | (10- 10.5) | 10.4 | (10.2- 10.6) | 8.0 | | (7.4- 8.6) | 8.5 | (7.9- 9) |  |
| Hamadan | 11.1 | (10.8- 11.4) | 12.5 | (12.0-13.0) | 9.9 | (9.6- 10.1) | 10.1 | (9.6- 10.6) | 7.3 | | (6.4- 8.8) | 6.3 | (5.1- 7.5) |  |
| ChaharM&Bakhtiari | 9.8 | (9.7- 10.0) | 10.4 | (10.2-10.7) | 10.0 | (9.9- 10.1) | 9.9 | (9.7- 10.1) | 8.8 | | (8.4- 9.2) | 7.3 | (6.7- 7.9) |  |
| Lorestan | 10.5 | (10.3- 10.8) | 11.5 | (11.2-11.8) | 9.6 | (9.3- 9.8) | 10.2 | (9.9- 10.5) | 7.2 | | (6.3- 8.8) | 7.5 | (6.7- 8.3) |  |
| Ilam | 10.4 | (10.1- 10.6) | 11.3 | (10.9-11.7) | 9.1 | (8.9- 9.4) | 9.4 | (9.0- 9.7) | 7.0 | | (6.4- 7.5) | 7.2 | (6.4- 8.0) |  |
| Kohgiluyeh&BoyerA | 11.1 | (10.7- 11.5) | 12.5 | (11.9-13.1) | 9.3 | (9.0- 9.7) | 9.9 | (9.4- 10.4) | 6.0 | | (5.0- 6.9) | 4.9 | (3.5- 6.2) |  |
| Bushehr | 10.5 | (10.1- 11.0) | 11.6 | (11-12.2) | 8.4 | (8.0- 8.9) | 8.7 | (8.1- 9.3) | 4.4 | | (3.1- 5.6) | 3.6 | (2.2- 5.0) |  |
| Zanjan | 11.2 | (10.9- 11.5) | 12.2 | (11.8-12.6) | 9.9 | (9.6- 10.2) | 10.4 | (10.0- 10.8) | 6.6 | | (5.7- 7.5) | 6.8 | (5.8- 7.8) |  |
| Semnan | 9.7 | (9.5- 9.8) | 9.7 | (9.6-9.8) | 9.5 | (9.4- 9.7) | 9.6 | (9.5- 9.7) | 10.9 | | (10.5- 11.3) | 10.5 | (10.3- 10.8) |  |
| Yazd | 9.3 | (9.1- 9.4) | 10.5 | (10.3-10.6) | 9.4 | (9.3- 9.6) | 10 | (9.8- 10.1) | 11.2 | | (10.7- 11.7) | 10.9 | (10.5- 11.3) |  |
| Hormozgan | 9.0 | (8.8- 9.2) | 10.2 | (10-10.5) | 8.1 | (7.9- 8.3) | 9.2 | (9.0- 9.4) | 6.2 | | (5.8- 6.7) | 7.2 | (6.7- 7.8) |  |
| Tehran | 9.2 | (9.2- 9.3) | 10.1 | (10-10.2) | 9.2 | (9.1- 9.2) | 9.7 | (9.6- 9.8) | 8.6 | | (8.5- 8.8) | 8.6 | (8.3- 8.8) |  |
| Ardabil | 9.8 | (9.6- 9.9) | 9.6 | (9.4-9.8) | 9.2 | (9.1- 9.4) | 9.2 | (9.0- 9.4) | 7.5 | | (7.0- 7.9) | 6.9 | (6.3- 7.5) |  |
| Qom | 7.3 | (7.2- 7.5) | 8.9 | (8.7-9.0) | 6.9 | (6.8- 7) | 8.2 | (8.1- 8.4) | 5.5 | | (5.1- 5.8) | 6.9 | (6.5- 7.3) |  |
| Qazvin | 10.4 | (10.1- 10.7) | 11.2 | (11-11.4) | 9.2 | (8.9- 9.4) | 10.1 | (9.9- 10.3) | 6.6 | | (5.8- 7.3) | 8.1 | (7.5- 8.6) |  |
| Golestan | 8.9 | (8.8- 9.1) | 9.8 | (9.8-9.9) | 9.7 | (9.6- 9.8) | 10.1 | (10.0- 10.1) | 10.1 | | (9.8- 10.4) | 10.5 | (10.4- 10.7) |  |
| Khorasan_North | 8.6 | (8.4- 8.9) | 9.8 | (9.6-10.0) | 9.8 | (9.6- 10.1) | 10.7 | (10.6- 10.9) | 11.8 | | (11.2- 12.4) | 12.2 | (11.8- 12.7) |  |
| Khorasan_South | 8.8 | (8.7- 8.9) | 10.3 | (10.1-10.5) | 9.1 | (9.0- 9.2) | 10.0 | (9.8- 10.2) | 8.4 | | (8.1- 8.8) | 8.2 | (7.6- 9.0) |  |
| Alborz | 8.9 | (8.6- 9.1) | 10.0 | (9.9-10.2) | 9.5 | (9.3- 9.7) | 10.2 | (10.0- 10.3) | 11.7 | | (11.1- 12.3) | 11.6 | (11.2- 12.0) |  |
| **Iran** | **9.6** | **(9.4- 9.9)** | **10.6** | **(10.3-10.8)** | **9.2** | **(9- 9.4)** | **9.8** | **(9.6- 10.0)** | **8.2** | | **(7.6- 8.8)** | **8.3** | **(7.7- 8.9)** |  |

| Table 6. Age standardized prevalence of hypertension at three time periods in national and subnational levels | | | | | | | | | | | | |
| --- | --- | --- | --- | --- | --- | --- | --- | --- | --- | --- | --- | --- |
| province | 2001 | | | | 2016 | | | | 2030 | | | |
|  | Female | | Male | | Female | | Male | | Female | | Male | |
|  | P | 95%CI | P | 95%CI | P | 95%CI | P | 95%CI | P | 95%CI | P | 95%CI |
| Markazi | 15.5 | (12.6-18.3) | 13.8 | (11.7-15.6) | 29.8 | (27.5-32.0) | 24.2 | (22.4- 25.9) | 42.0 | (38.4- 46.0) | 34.8 | (31.2-38.5) |
| Gilan | 18.8 | (14.4-23) | 14.9 | (12.1-18.4) | 38.5 | (35.5-41.4) | 28.6 | (26.3- 30.8) | 54.1 | (49.8- 58.6) | 41.5 | (37.4-45.7) |
| Mazandaran | 18.3 | (15.1-21.5) | 15.0 | (12.8-18.1) | 34.9 | (32.5-37.3) | 25.7 | (24.0- 27.4) | 47.7 | (44.3- 51.1) | 35.3 | (32.6-38.4) |
| Azerbaijan_East | 19.8 | (16.9-22.6) | 16.5 | (14.2-19.9) | 34.6 | (32.4-36.8) | 27.9 | (26.0- 29.7) | 46.4 | (43.2- 50.0) | 38.5 | (35.2-42.1) |
| Azerbaijan_West | 18.3 | (15.3-21.3) | 15.7 | (13.6-18.3) | 33.6 | (31.3-35.9) | 26.2 | (24.5- 27.9) | 45.9 | (42.5- 49.7) | 35.6 | (32.9-38.6) |
| Kermanshah | 17.9 | (14.5-21.2) | 15.2 | (12.7-17.5) | 34.2 | (31.7-36.7) | 26.8 | (24.8- 28.7) | 47.5 | (43.9- 51.4) | 37.7 | (34.4-41.2) |
| Khuzestan | 16.5 | (13.4-19.5) | 15.1 | (12.9-16.4) | 31.5 | (29.1-33.8) | 25.9 | (24.1- 27.7) | 44.0 | (40.5- 47.9) | 36.2 | (32.7-40.5) |
| Fars | 16.0 | (14.0-18.0) | 13.5 | (11.6-118) | 27.0 | (25.3-28.6) | 23.0 | (21.5- 24.5) | 36.0 | (33.0- 38.9) | 31.6 | (29.1-34.4) |
| Kerman | 16.1 | (14.2-18.1) | 13.5 | (12.1-16.2) | 26.8 | (25.1-28.4) | 20.9 | (19.7- 22.1) | 35.6 | (33.2- 38.2) | 27.7 | (25.7-30.2) |
| Khorasan_Razavi | 16.2 | (13.6-18.7) | 15.2 | (13.1-16.6) | 29.6 | (27.5-31.6) | 26.1 | (24.3- 27.8) | 40.7 | (37.6- 44.0) | 35.8 | (33.0-39.0) |
| Isfahan | 15.5 | (13.1-17.9) | 13.1 | (10.9-15.1) | 28.1 | (26.2-30.0) | 23.8 | (22.0- 25.5) | 38.7 | (35.7- 41.8) | 33.5 | (30.7-36.5) |
| Sistan&Baluchistan | 12.1 | (10-14.2) | 11.2 | (9.6-12.14) | 22.5 | (20.7-24.2) | 19.3 | (17.9- 20.7) | 32.2 | (29.2- 35.7) | 27.1 | (24.8-29.7) |
| Kurdistan | 16.2 | (12.5-19.8) | 14.3 | (11.7-16.5) | 33.6 | (30.9-36.2) | 26.6 | (24.5- 28.6) | 48.1 | (44.0- 52.3) | 38.0 | (34.6-41.5) |
| Hamadan | 17.6 | (14.2-21) | 14.7 | (12.7-17.2) | 34.8 | (32.3-37.4) | 24.6 | (23.0- 26.2) | 48.3 | (44.6- 52.2) | 33.8 | (31.0-36.9) |
| ChaharM&Bakhtiari | 19.0 | (15.9-22.1) | 16.9 | (14.9-19.0) | 34.7 | (32.3-37.1) | 27.5 | (25.7- 29.1) | 47.4 | (43.9- 51.0) | 36.9 | (34.0-40.3) |
| Lorestan | 16.9 | (12.9-20.7) | 15.2 | (13.0-16.9) | 33.9 | (31.1-36.6) | 25.3 | (23.5- 27.0) | 48.3 | (43.9- 53.0) | 35.3 | (32.0-39.0) |
| Ilam | 13.3 | (9.5-16.9) | 12.3 | (10.1-13.5) | 29.9 | (27.1-32.5) | 23.2 | (21.3- 25.0) | 44.8 | (40.0- 49.4) | 33.3 | (30.0-36.9) |
| Kohgiluyeh&BoyerAhmad | 17.2 | (14.2-20.1) | 14.5 | (12.3-17.2) | 31.6 | (29.4-33.9) | 25.1 | (23.3- 26.8) | 43.9 | (40.5- 47.6) | 35.2 | (32.0-38.7) |
| Bushehr | 18.6 | (16.4-20.7) | 15.3 | (13.7-18.4) | 30.2 | (28.4-32.0) | 23.3 | (22.0- 24.7) | 39.2 | (36.0- 42.0) | 30.8 | (28.5-33.3) |
| Zanjan | 17.9 | (15.1-20.7) | 15.5 | (13.7-17.1) | 32.6 | (30.4-34.8) | 25.1 | (23.6- 26.6) | 44.3 | (41.0- 47.7) | 33.6 | (31.1-36.5) |
| Semnan | 17.0 | (14.1-19.9) | 15.5 | (13.1-11.0) | 31.7 | (29.4-33.9) | 27.6 | (25.6- 29.5) | 43.5 | (40.3- 46.9) | 38.2 | (35.0-41.8) |
| Yazd | 13.7 | (11.7-15.7) | 11.4 | (9.6-13.7) | 24.6 | (22.9-26.3) | 20.6 | (19.1- 22.0) | 33.6 | (30.9- 36.2) | 29.1 | (26.5-31.9) |
| Hormozgan | 14.9 | (12.5-17.4) | 13.4 | (11.2-14.5) | 27.9 | (25.9-29.8) | 24.2 | (22.4- 25.9) | 38.5 | (35.6- 41.6) | 34.0 | (30.9-37.4) |
| Tehran | 15.6 | (13.6-17.7) | 14.8 | (12.6-15.6) | 26.4 | (24.6-28.1) | 25.8 | (24.0- 27.6) | 35.5 | (32.5- 38.6) | 35.8 | (32.8-39.1) |
| Ardabil | 18.3 | (14.9-21.7) | 16.2 | (13.6-18.9) | 35.0 | (32.5-37.5) | 27.9 | (25.8- 29.8) | 48.6 | (44.7- 52.7) | 40.0 | (36.1-43.9) |
| Qom | 15.5 | (12.8-18.1) | 15.0 | (12.5-15.8) | 28.9 | (26.8-31.0) | 26.8 | (24.8- 28.7) | 40.2 | (37.0- 43.8) | 37.9 | (34.4-41.6) |
| Qazvin | 15.3 | (12.7-17.9) | 14.0 | (12.0-15.7) | 28.9 | (26.8-30.9) | 23.9 | (22.2- 25.5) | 40.1 | (36.9- 43.6) | 33.3 | (30.3-36.5) |
| Golestan | 15.6 | (12.6-18.6) | 12.5 | (10.2-15.6) | 30.3 | (27.9-32.6) | 23.2 | (21.3- 25.0) | 43.0 | (39.2- 47.1) | 34.7 | (30.9-38.2) |
| Khorasan_North | 19.3 | (15.9-22.7) | 17.2 | (14.7-19.9) | 36.6 | (33.9-39.1) | 29.8 | (27.8- 31.8) | 50.1 | (46.1- 54.3) | 40.8 | (37.5-44.6) |
| Khorasan_South | 15.2 | (12.2-18.1) | 14.0 | (12.1-15.2) | 29.0 | (26.7-31.2) | 23.7 | (22.1- 25.3) | 41.4 | (37.8- 45.2) | 32.6 | (30.1-35.1) |
| Alborz | 24.7 | (22.6-26.7) | 22.4 | (20.4-24.6) | 34.9 | (33.3-36.6) | 32.2 | (30.6- 33.8) | 40.2 | (36.7- 43.7) | 38.5 | (34.7-41.8) |
| **Iran** | **16.9** | **(14.1-19.7)** | **15.0** | **(12.9-16.1)** | **30.9** | **(28.7-33.1)** | **25.5** | **(23.8- 27.2)** | **42.5** | **(39- 46.1)** | **35.2** | **(32.1-38.5)** |

| Table 7. Age standardized prevalence of obesity and overweight at three time periods in national and subnational levels | | | | | | | | | | | | |
| --- | --- | --- | --- | --- | --- | --- | --- | --- | --- | --- | --- | --- |
| province | 2001 | | | | 2016 | | | | 2030 | | | |
|  | Female | | Male | | Female | | Male | | Female | | Male | |
|  | P | 95%CI | P | 95%CI | P | 95%CI | P | 95%CI | P | 95%CI | P | 95%CI |
| Markazi | 18.2 | (14.1- 22.0) | 9.6 | (7.4-11.6) | 34.9 | (32.7- 37.0) | 19.3 | (17.7-20.8) | 44.9 | (42.3-47.5) | 27.9 | (25.2- 30.8) |
| Gilan | 20.3 | (16.2- 23.9) | 10.9 | (8.8-13) | 36.6 | (34.4- 38.5) | 20.9 | (19.3-22.4) | 45.9 | (43.7-48.2) | 29.4 | (26.7- 32.1) |
| Mazandaran | 23.0 | (18.7- 26.9) | 13.1 | (10.7-15.4) | 40.0 | (37.9- 41.9) | 23.9 | (22.3-25.5) | 49.0 | (46.9-50.9) | 32.4 | (30.0- 34.9) |
| Azerbaijan_East | 19.6 | (15.5- 23.3) | 10.7 | (8.5-12.8) | 36.1 | (33.9- 38.1) | 20.5 | (18.9-22.0) | 45.7 | (43.2-48.2) | 28.9 | (26.4- 31.5) |
| Azerbaijan_West | 20.3 | (15.8- 24.3) | 10.9 | (8.5-13.3) | 37.5 | (35.3- 39.6) | 21.6 | (19.9-23.2) | 47.2 | (44.8-49.7) | 30.2 | (27.7- 33.0) |
| Kermanshah | 18.4 | (14.6- 22.0) | 9.9 | (7.7-11.9) | 34.5 | (32.4- 36.4) | 19.4 | (17.9-20.9) | 43.9 | (41.6-46.2) | 27.6 | (25.2- 30.1) |
| Khuzestan | 18.5 | (14.6- 22.1) | 9.9 | (7.8-11.9) | 34.4 | (32.3- 36.4) | 19.3 | (17.7-20.8) | 44.0 | (41.6-46.5) | 27.7 | (24.9- 31.1) |
| Fars | 15.0 | (11.7- 18.2) | 7.8 | (6.1-9.3) | 29.5 | (27.4- 31.4) | 15.2 | (14.0-16.4) | 39.2 | (36.3-42.5) | 22.0 | (19.9- 24.5) |
| Kerman | 12.9 | (9.9- 15.8) | 6.4 | (4.9-7.9) | 26.1 | (24.2- 28.0) | 13.1 | (12.0-14.3) | 35.6 | (33.1-38.4) | 19.8 | (17.5- 22.1) |
| Khorasan_Razavi | 15.7 | (12.0- 19.2) | 8.1 | (6.2-9.9) | 30.9 | (28.7- 32.9) | 16.6 | (15.2-18.0) | 41.0 | (38.2-43.9) | 24.5 | (22.1- 27.0) |
| Isfahan | 18.3 | (14.5- 21.7) | 9.6 | (7.5-11.6) | 33.7 | (31.6- 35.6) | 18.8 | (17.3-20.2) | 43.2 | (40.8-45.8) | 26.9 | (24.5- 29.5) |
| Sistan&Baluchistan | 10.7 | (8.0- 13.3) | 5.1 | (3.7-6.4) | 22.5 | (20.6- 24.2) | 11.0 | (9.9-12.0) | 31.9 | (29.1-34.7) | 17.0 | (15.1- 19.0) |
| Kurdistan | 17.5 | (13.4- 21.3) | 9.1 | (7.1-11.1) | 34.0 | (31.8- 36.1) | 18.4 | (16.9-19.8) | 44.1 | (41.5-46.6) | 26.3 | (24.0- 28.6) |
| Hamadan | 16.6 | (12.7- 20.2) | 8.8 | (6.9-10.7) | 32.7 | (30.5- 34.7) | 17.5 | (16.1-18.8) | 42.7 | (40.1-45.4) | 25.2 | (22.9- 27.7) |
| ChaharM&Bakhtiari | 16.9 | (13.1- 20.4) | 8.7 | (6.7-10.6) | 32.4 | (30.3- 34.4) | 17.6 | (16.2-19) | 42.3 | (39.7-44.9) | 25.6 | (23.0- 28.3) |
| Lorestan | 16.3 | (12.3- 20.0) | 8.6 | (6.6-10.6) | 32.4 | (30.2- 34.4) | 17.6 | (16.1-19) | 42.2 | (39.7-44.7) | 25.7 | (23.2- 28.5) |
| Ilam | 14.3 | (10.9- 17.5) | 7.5 | (5.8-9.1) | 28.7 | (26.6- 30.6) | 15.1 | (13.8-16.3) | 38.3 | (35.8-40.9) | 22.2 | (19.8- 24.6) |
| Kohgiluyeh&BoyerA | 18.0 | (14.1- 21.6) | 9.3 | (7.2-11.3) | 34.2 | (32.1- 36.2) | 19.1 | (17.6-20.6) | 43.8 | (41.5-46.2) | 27.3 | (24.8- 30.0) |
| Bushehr | 15.8 | (12.4- 18.9) | 8.1 | (6.4-9.8) | 30.3 | (28.2- 32.2) | 16.2 | (14.9-17.5) | 39.9 | (37.3-42.6) | 23.7 | (21.3- 26.0) |
| Zanjan | 17.6 | (13.5- 21.3) | 9.3 | (7.3-11.3) | 34.0 | (31.8- 36.1) | 18.7 | (17.2-20.2) | 44.0 | (41.4-46.7) | 27.1 | (24.4- 29.8) |
| Semnan | 19.0 | (15.2- 22.6) | 9.8 | (7.7-11.9) | 34.8 | (32.7- 36.8) | 19.5 | (18.0-21.0) | 44.4 | (42.0-46.8) | 27.9 | (25.3- 30.8) |
| Yazd | 18.5 | (14.8- 21.9) | 10.0 | (7.9-11.9) | 34.0 | (31.9- 35.9) | 19.0 | (17.5-20.4) | 43.4 | (41.1-45.9) | 27.0 | (24.5- 29.5) |
| Hormozgan | 13.0 | (10.3- 15.5) | 6.2 | (4.8-7.6) | 25.5 | (23.7- 27.1) | 12.9 | (11.8-14.0) | 34.2 | (31.9-36.6) | 19.2 | (17.2- 21.5) |
| Tehran | 20.7 | (17.1- 23.9) | 11.5 | (9.5-13.4) | 36.1 | (34.1- 37.9) | 20.7 | (19.2-22.1) | 45.1 | (42.7-47.6) | 28.5 | (26.2- 31.0) |
| Ardabil | 22.0 | (18.0- 25.6) | 12.2 | (9.7-14.5) | 38.2 | (36.2- 40.1) | 22.9 | (21.2-24.5) | 47.3 | (45.1-49.6) | 31.7 | (29.1- 34.3) |
| Qom | 20.4 | (16.3- 24.2) | 11.2 | (8.8-13.5) | 37.0 | (34.8- 38.9) | 21.7 | (20.0-23.3) | 46.5 | (44.1-48.9) | 30.6 | (27.9- 33.4) |
| Qazvin | 18.6 | (14.6- 22.2) | 9.8 | (7.7-11.8) | 34.7 | (32.5- 36.7) | 19.4 | (17.8-20.9) | 44.6 | (42.0-47.3) | 28.0 | (25.2- 30.7) |
| Golestan | 18.9 | (14.2- 23.1) | 11.9 | (9.6-14) | 36.9 | (34.5- 39.0) | 21.9 | (20.3-23.4) | 47.0 | (44.4-49.5) | 30.4 | (27.9- 33.0) |
| Khorasan_North | 16.1 | (12.6- 19.3) | 7.7 | (5.8-9.7) | 30.8 | (28.7- 32.8) | 16.9 | (15.4-18.3) | 40.8 | (38.0-43.7) | 25.2 | (22.5- 27.9) |
| Khorasan_South | 13.1 | (9.8- 16.0) | 6.2 | (4.5-7.7) | 25.9 | (23.9- 27.8) | 13.2 | (12.0-14.4) | 35.8 | (33.0-38.5) | 20.0 | (18.0- 21.9) |
| Alborz | 20.8 | (17.3- 24.0) | 10.6 | (8.3-12.7) | 35.5 | (33.6- 37.3) | 20.9 | (19.3-22.4) | 44.4 | (42.2-46.7) | 29.4 | (26.9- 32.1) |
| **Iran** | **17.6** | **(13.8- 21.0)** | **9.3** | **(7.3-11.2)** | **33.1** | **(31.0- 35.0)** | **18.3** | **(16.9-19.8)** | **42.6** | **(40.2-45.2)** | **26.3** | **(23.8- 28.9)** |

| Table 8. Age standardized prevalence of diabetes at three time periods in national and subnational levels | | | | | | | | | | | | | |
| --- | --- | --- | --- | --- | --- | --- | --- | --- | --- | --- | --- | --- | --- |
| province | 2001 | | | | 2016 | | | | 2030 | | | | |
|  | Female | | Male | | Female | | Male | | Female | | Male | |  |
|  | P | 95%CI | P | 95%CI | P | 95%CI | P | 95%CI | P | 95%CI | P | 95%CI |  |
| Markazi | 7.2 | (6.7- 7.6) | 5.8 | (5.4- 6.3) | 10.0 | (9.6- 10.4) | 8.7 | (8.3- 9.2) | 12.5 | (12.0- 12.9) | 11.1 | (10.7-11.6) |  |
| Gilan | 7.5 | (7.0- 8.0) | 5.9 | (5.4- 6.3) | 10.1 | (9.7- 10.5) | 8.6 | (8.2- 9.0) | 12.7 | (12.2- 13.1) | 11.2 | (10.8-11.6) |  |
| Mazandaran | 9.6 | (9.1- 10.0) | 7.8 | (7.4- 8.3) | 11.3 | (10.8- 11.8) | 9.2 | (8.8- 9.7) | 12.8 | (12.2- 13.2) | 10.4 | (9.8-10.9) |  |
| Azerbaijan_East | 8.2 | (7.8- 8.6) | 6.7 | (6.3- 7.1) | 10.2 | (9.8- 10.6) | 8.6 | (8.2- 9.0) | 12.0 | (11.6- 12.4) | 10.3 | (9.9-10.7) |  |
| Azerbaijan_West | 7.3 | (6.8- 7.7) | 5.8 | (5.4- 6.2) | 9.8 | (9.3- 9.2) | 8.0 | (7.6- 8.4) | 12.1 | (11.6- 12.5) | 9.7 | (9.3-10.1) |  |
| Kermanshah | 7.7 | (7.3- 8.1) | 6.2 | (5.8- 6.6) | 10.1 | (9.6- 10.5) | 8.1 | (7.7- 8.5) | 12.2 | (11.7- 12.6) | 9.7 | (9.2-10.1) |  |
| Khuzestan | 10.1 | (9.7- 10.5) | 8.3 | (7.9- 8.7) | 12.0 | (11.6- 12.4) | 9.9 | (9.5- 10.4) | 13.5 | (13.0- 13.9) | 11.1 | (10.6-11.5) |  |
| Fars | 8.2 | (7.8- 8.6) | 6.6 | (6.2- 7.0) | 9.9 | (9.5- 9.3) | 8.1 | (7.7- 8.5) | 11.4 | (11.0- 11.8) | 9.3 | (8.9-9.8) |  |
| Kerman | 6.6 | (6.2- 7.7) | 5.5 | (5.1- 6.0) | 7.7 | (7.2- 7.1) | 6.5 | (6- 6.9) | 8.3 | (7.9- 8.9) | 6.9 | (6.4-7.4) |  |
| Khorasan_Razavi | 6.4 | (6.1- 6.9) | 5.3 | (4.9- 5.7) | 8.6 | (8.2- 8.9) | 7.1 | (6.7- 7.6) | 10.4 | (10- 10.8) | 8.5 | (8.1-8.9) |  |
| Isfahan | 9.8 | (9.2- 10.2) | 8.1 | (7.6- 8.6) | 12.0 | (11.5- 12.5) | 10.3 | (9.8- 10.7) | 14.3 | (13.8- 14.8) | 12.3 | (11.8-12.7) |  |
| Sistan&Baluchistan | 6.3 | (5.8- 6.8) | 5.2 | (4.7- 5.6) | 8.5 | (8- 8.9) | 6.8 | (6.3- 7.3) | 10.4 | (9.9- 10.9) | 8.1 | (7.5-8.6) |  |
| Kurdistan | 6.3 | (5.9- 6.8) | 5.0 | (4.6- 5.4) | 9.4 | (8.9- 9.9) | 7.4 | (7- 7.8) | 12.1 | (11.6- 12.6) | 9.3 | (8.9-9.8) |  |
| Hamadan | 6.5 | (6.1- 6.9) | 5.3 | (4.8- 5.7) | 8.6 | (8.2- 8.9) | 6.9 | (6.5- 7.4) | 10.3 | (9.9- 10.7) | 8.2 | (7.7-8.6) |  |
| ChaharM&Bakhtiari | 6.9 | (6.5- 7.4) | 5.3 | (5.0- 5.8) | 8.4 | (7.9- 8.8) | 7.0 | (6.6- 7.4) | 9.5 | (9.1- 10) | 8.3 | (7.9-8.8) |  |
| Lorestan | 6.6 | (6.2- 7.1) | 5.3 | (4.9- 5.7) | 8.6 | (8.1- 8.9) | 7.1 | (6.7- 7.5) | 10.3 | (9.9- 10.8) | 8.5 | (8.1-9.0) |  |
| Ilam | 6.0 | (5.6- 6.5) | 4.7 | (4.2- 5.1) | 8.1 | (7.7- 8.5) | 6.6 | (6.2- 7.0) | 9.9 | (9.4- 10.3) | 8.2 | (7.7-8.6) |  |
| Kohgiluyeh&BoyerA | 4.9 | (4.5- 5.4) | 3.7 | (3.3- 4.2) | 8.0 | (7.5- 8.4) | 6.3 | (5.8- 6.7) | 10.8 | (10.3- 11.2) | 8.4 | (8-8.9.0) |  |
| Bushehr | 7.0 | (6.6- 7.4) | 5.3 | (4.9- 5.7) | 8.4 | (8- 8.8) | 7.5 | (7.1- 7.9) | 9.6 | (9.2- 10.1) | 9.9 | (9.4-10.4) |  |
| Zanjan | 6.2 | (5.8- 6.6) | 4.9 | (4.5- 5.4) | 8.7 | (8.2- 8.1) | 7.4 | (6.9- 7.8) | 10.6 | (10.1- 11.2) | 9.2 | (8.6-9.7) |  |
| Semnan | 9.6 | (9.1- 10.0) | 8.2 | (7.7- 8.6) | 11.1 | (10.7- 11.6) | 9.8 | (9.4- 10.2) | 12.3 | (11.9- 12.8) | 11.2 | (10.7-11.6) |  |
| Yazd | 11 | (10.5- 11.4) | 9.2 | (8.8- 9.6) | 12.5 | (12- 12.9) | 10.4 | (9.9- 10.8) | 13.6 | (13.2- 14.1) | 11.1 | (10.6-11.5) |  |
| Hormozgan | 5.7 | (5.3- 6.2) | 4.6 | (4.1- 5.0) | 7.6 | (7.2- 7.1) | 6.2 | (5.8- 6.7) | 9.3 | (8.8- 9.8) | 7.5 | (7.0- 8.0) |  |
| Tehran | 9.2 | (8.6- 9.9) | 7.5 | (6.9- 8.1) | 10.5 | (10- 10.11) | 9.1 | (8.6- 9.6) | 11.6 | (11.1- 12.1) | 10.6 | (10.1-11.1) |  |
| Ardabil | 6.6 | (6.1- 7.7) | 5.2 | (4.7- 5.6) | 9.1 | (8.7- 9.5) | 7.5 | (7.0- 7.9) | 11.1 | (10.7- 11.5) | 9.4 | (8.9-9.8) |  |
| Qom | 11.6 | (11.0- 12.0) | 9.4 | (8.7- 9.9) | 13.1 | (12.5- 13.7) | 10.8 | (10.3- 11.4) | 14.3 | (13.8- 14.8) | 11.8 | (11.3-12.3) |  |
| Qazvin | 8.3 | (8.0- 8.8) | 7.0 | (6.6- 7.4) | 10.8 | (10.4- 10.2) | 9.5 | (9.1- 9.9) | 13 | (12.6- 13.4) | 11.7 | (11.2-12.2) |  |
| Golestan | 7.3 | (6.9- 7.8) | 5.7 | (5.3- 6.2) | 9.1 | (8.6- 9.6) | 7.1 | (6.6- 7.5) | 10.7 | (10.3- 11.2) | 8.1 | (7.6-8.6) |  |
| Khorasan_North | 5.6 | (5.1- 6.1) | 4.7 | (4.2- 5.1) | 7.7 | (7.3- 7.2) | 6.5 | (6.0- 6.9) | 9.7 | (9.2- 10.2) | 7.9 | (7.4-8.4) |  |
| Khorasan_South | 5.7 | (5.3- 6.1) | 4.7 | (4.3- 5.2) | 7.7 | (7.3- 7.2) | 6.2 | (5.7- 6.7) | 9.5 | (9.0- 9.9) | 7.4 | (6.9-7.8) |  |
| Alborz | 10.4 | (9.9- 10.9) | 8.5 | (8.0- 9.1) | 11.7 | (11.2- 11.2) | 10.4 | (9.9- 10.9) | 12.8 | (12.3- 13.3) | 12.3 | (11.7-12.8) |  |
| **Iran** | **7.6** | **(7.2- 8.1)** | **6.2** | **(5.7- 6.7)** | **9.6** | **(9.1- 9.10)** | **8** | **(7.6- 8.5)** | **11.2** | **(10.8- 11.7)** | **9.5** | **(9.0-10)** |  |
